# Midgestational injection of highly expanded human CD34+ cells increases lineages of human immune cells and supports thymic development in *RAG2-/-IL2RG-/Y* SCID pigs

**DOI:** 10.3389/fimmu.2026.1751541

**Published:** 2026-03-11

**Authors:** Ahlea M. Forster, Amanda Ahrens Kress, Matti Kiupel, Joan Cunnick, Dennis A. Webster, Jarryd M. Campbell, Adrienne L. Watson, Ohad Gafni, Daniel F. Carlson, Branden S. Moriarity, Beau R. Webber, Brett Napiwocki, Lance Daharsh, Jason W. Ross, Mary B. Sauer, Christopher K. Tuggle

**Affiliations:** 1 Department of Animal Science, Iowa State University, Ames, IA, United States; 2 Laboratory Animal Resources, Iowa State University, Ames, IA, United States; 3 Department of Pathobiology and Diagnostic Investigation, College of Veterinary Medicine, Michigan State University, East Lansing, MI, United States; 4 Recombinetics, Inc., New Brighton, MN, United States; 5 Department of Pediatrics, University of Minnesota, Minneapolis, MN, United States

**Keywords:** biomedical model development, humanization, SCID-severe combined immunodeficiency, swine, translational biomedicine

## Abstract

Severe combined immunodeficiency (SCID) pigs have become a promising large animal model for biomedical research, offering significant advantages over traditional mouse models due to their anatomical, physiological, and genetic similarities to humans. Humanized SCID pig models can potentially improve preclinical research in areas such as cancer immunotherapies, stem cell therapies, and transplantation methods, yet often lack significant lymphocyte development, including evidence of B cell and myeloid cell development. This work aims to increase the extent of humanization of the SCID pig. CRISPR guide RNAs were successfully developed for the RAG2 and IL2RG genes, and a double-knockout cell line (RAG2-/-IL2RG-/Y, RG) was established. Somatic cell nuclear transfer (SCNT) was then used to create cloned SCID fetuses, which were injected intraperitoneally with *in vitro* expanded human CD34+ umbilical cord cells at day 41–42 of gestation. Human leukocytes, including T, B, NK, and myeloid cell types, were detected in peripheral blood, spleen, bone marrow and within the thymus of neonatal animals using flow cytometry. Six of the twelve pigs injected had >5% human cells within the CD45+ cell thymic population. Histology of thymus tissues from multiple pigs showed substantial development of the cortex and medulla, which is absent in non-injected RG neonates. This work demonstrates an improvement in the spectrum of xenogenic immune cell lineages developed using an RG line injected with highly expanded CD34+ cells, yet functional analysis of these cell types is needed for further establishment of an *in utero* humanized SCID pig model.

## 1 Introduction

Biomedical research has long sought animal models that closely mimic human physiology and disease processes. Mouse models that can fully engraft human cells using an injection of human PBMCs, human stem cells, or fetal bone marrow (BM), liver, and thymus cells allow researchers to study human biomedical questions (1–3). While mouse models have been invaluable in advancing our understanding of biological mechanisms, they often fall short in translating findings to human applications due to the significant differences in size, lifespan, and physiology, with significant differences in including immunology (4, 5). In recent years, pigs have emerged as a promising large animal model, offering a closer relation to human biology in many aspects (6–8).

SCID pigs represent a breakthrough in this area of research. These animals lack a functional adaptive immune system, providing a unique platform for studying human diseases and testing novel therapies by minimizing rejection by the host immune system. One strain of naturally occurring SCID pig with a spontaneous mutation in the DCLRE1C gene has been reported (9, 10). Further development of SCID pigs has been made possible through advances in genetic engineering techniques, including CRISPR/Cas9 technology to target specific immune-related gene combinations, including DCLRE1C and IL2RG (11), as well as several reports targeting RAG1 or RAG2 in combination with IL2RG (8, 12; reviewed in 7) to remove most if not all (13), adaptive immune function and maximize humanization levels.

By engrafting human hematopoietic stem cells (hHSC) into SCID pigs, researchers aim to recapitulate the human immune cell repertoire and generate an immune response representative of the response seen in humans, in a physiologically relevant large animal model (7, 8, 11, 12). This approach holds immense potential for studying human-specific diseases, testing immunotherapies, and advancing our understanding of complex immune interactions that are difficult in traditional model systems. The first report of SCID pig humanization used a DCLRE1C-/-; IL2RG-/Y model with a laparotomy-based fetal stage injection of hHSC and showed low to moderate levels of human T cells in blood and thymus, as well as a few B cells in blood, spleen and mesenteric lymph node in neonatal pigs. More recently, Hu et al. described the creation of a RAG1-/-;IL2RG-/Y double mutant (RG), as well as a triple mutant containing a further mutation in the CD47 gene (RGD) and reported improved humanization levels in post-natal cell administration procedures in the RDG mutant line as compared to their RG line (12, 14). Specifically, they found substantial levels of B and T cells in thymus, spleen and BM, as well as lower levels of myeloid and NK cells, which is consistent with results observed for humanization of SCID mice (15). In these humanized pigs, they documented circulating human immunoglobulins to show human B cell function and used *in vitro* stimulation with CD3/CD28 to show T cell function. They also used single-cell RNAseq analysis of human cell populations in thymus and spleen to determine cell types developing in these pigs, both at the transcriptome level and T and B cell receptor repertoires (12).

However, Hu et al. exclusively used post-natal administration of the CD34+ hHSC, requiring maintenance of disease-susceptible pigs for several months while the pig matures and the human immune system develops. To test whether earlier administration (prior to full immune tissue development) of a greatly expanded cell dose could be effective, we tested several adjustments to our previously published work. Fetal liver injection surgeries previously described (11), were performed at day 41 gestation with 10-20-fold more cells that were expanded using improved methods resulting in higher CD34+ fractions in the injected cell population. Flow cytometry analysis of animals at postnatal day 0 showed large T cell populations in samples from pigs injected with human cells. Histology of thymic tissue showed a more developed structure in the injected animals compared to those that had not been injected. Other tissues such as spleen and BM contained human CD45+ cell subsets (T and B cells) that were fewer in number than the thymus tissue, yet the spleen had improved structural appearance compared to the non-injected animals. This successfully established an improvement in prenatal humanization of the SCID pig model, demonstrating that expanded human CD34+ cells injected during the early stages of immune development, even in an RG model, leads to increased human immune cell differentiation and expansion in pig tissues and blood.

## 2 Methods and materials

### 2.1 Creation of animals through somatic cell nuclear transfer

The protocol for creating a new line of SCID pigs through mutagenesis and SCNT for use in humanization experiments is as follows:

#### 2.2.1 Editing reagents

Alt-R CRISPR-Cas9 crRNA, Alt-R CRISPR-Cas9 tracrRNA, single-stranded oligodeoxynucleotide (ssODN) homology-directed repair (HDR) templates and Cas9 protein (Alt-R S.p. HiFi Cas9 Nuclease V3) were purchased from Integrated DNA Technologies (Coralville, IA). Knockout ssODNs were designed to introduce a premature termination codon, frameshift mutation, and a novel HindIII restriction site for genotyping. See the sequences below in **Table 1**.

**Table 1:** Editing reagents.

Name	Reagent type	Sequence (5′-3′)
*ssIL2Rg* c 2.1	crRNA	GAAACGUUGAGAGUCCCAGG
*ssRAG2* c.1.1	crRNA	AUGUGCAAGUGGCUGGGUAG
*RAG2* c.1.1 HDR oligo HindIII	ssODN	CAGACTCTAAGCTGCTTTTGAATGTGCAAGTGGCTGGGTAGCGAAGCTTTAGAGAGGAGGAAGGTAGC AGGAATCCTTAGAGAAAAGTGC
*IL2Rg* c 2.1 HDR oligo HindIII	ssODN	GAACCTTTGGGAGGGGTAGAGTGGAAACGTTGAGAGTCCCAGGAAGCTTTAGGGTGTAGAGAGCAGGA GGAAATCTAGGACGGGGAGAAA

#### 2.2.2 Single cell derived clonal isolation

Primary Yorkshire fibroblast cells were maintained at 37 °C, 5% CO_2_ in DMEM supplemented with 10% fetal bovine serum and 100 I.U./mL penicillin and streptomycin. Ribonucleoprotein (RNP) complexes (120 pmol crRNA:tracrRNA complex, 17.2 µg Alt-R S.p. HiFi Cas9 Nuclease V3, and 0.2 nmol of ssODN) for each target were prepared immediately prior to transfection following the manufacturer’s recommendations. Electroporation of the cells was performed with the 100 µL Neon Transfection System (Invitrogen; Carlsbad, CA) using the following parameters: input voltage, 1800 V; pulse width, 20 ms; and pulse number, 1. Transfected cells were allowed to recover for three days prior to being seeded at low density, subcultured, and screened for HR-directed repair by a restriction length polymorphism (RFLP) assay.

#### 2.2.3 Mutation detection

Cell lysis was performed using 1X PCR compatible lysis buffer (10 mM Tris-Cl pH 8.0, 2 mM EDTA, 0.45% Tryton X-100 (vol/vol), 0.45% Tween-20 (vol/vol)) freshly supplemented with 200 μg/mL Proteinase K. The lysates were incubated in a thermal cycler using the following program: 55 °C for 60 minutes, 95 °C for 15 minutes. Identification of RAG2 and IL2Rg HDR was performed by PCR amplification using AccuStart II GelTrack PCR SuperMix (Quantabio; Beverly, MA) with 1 μL of the cell lysate as a template using the following primers and cycling programs in **Table 2**.

**Table 2:** Primers for single-cell clonal isolation.

Primer name	Sequence (5′-3′)	Cycling program
*ssIL2Rg* E2 NJ F1	CTCCCCCACTTCATTTTCTCCCC	1 cycle (95 °C, 2 minutes), 35 cycles of (95 °C, 20 s; 62 °C, 20 s; 72 °C, 40 s), 1 cycle (72 °C, 5 min)
*ssIL2Rg* E2 NJ R1	GATTCCACAGTCCAGCCTCAGCTC	
*ssRAG2* NJ F1	CCCAGCTGCCTGGATTTTTGC	1 cycle (95 °C, 2 minutes), 35 cycles of (95 °C, 20 s; 62 °C, 20 s; 72 °C, 40 s), 1 cycle (72 °C, 5 min)
*ssRAG2* NJ R1	CCGTCCTCCAAAGAGAACACCCA	

Amplicons were then digested with HindIII (New England Biolabs; Ipswich, MA) and visualized using agarose gel electrophoresis. Clones homozygous by RFLP were verified by Sanger sequencing (ACGT; Wheeling, IL) before somatic cell nuclear transfer (SCNT) was performed at Trans Ova Genetics (Sioux Center, IA). Initial litters of pigs created from these cells were genotyped to verify mutations in the RAG2 and IL2RG loci. Animals were confirmed pregnant before being transported to ISU for fetal liver injection surgeries and c-sections.

### 2.2 Expansion of cells

To test the effect of increasing injected cells to 20–40 million per injection, we expanded cells using a modification of the published protocol by Boitano et al. (16). Human CD34+ cells were obtained as purified CD34+ cells (StemCell Technologies/StemExpress). Umbilical cord blood (UCB)) CD34+ cells were expanded for 21 days in media containing StemSpan SFEM II (StemCell Technologies) in the presence of SCF 100 ng/mL (PeproTech), Flt3L 100 ng/mL (R&D Systems), IL-6–100 ng/mL (PeproTech), TPO 100 ng/mL (PeproTech), SR-1 750nM (Caymen Chemical), and UM729 500nM (StemCell Technologies) with half media changes every 3–4 days. The 21-day expansion routinely resulted in >100–200 fold more cells than input, without significant change to the fraction of HSC phenotype cells (**Supplementary Figure 1**). **Supplementary Table 1** which shows the calculations for HSC phenotype that are pictured in **Supplementary Figure 1** is included. Each tube for fetal implantation was prepared with either 25 million or 50 million cells, allowing for some loss of live cells and resulting a single injection of 20 million or 40 million live cells, respectively.

For some transplantations, a CAR-T transgene modified cell line was used. A high-density culture method was used for CD34+ cells transduced with lentivirus (LV) (17). Briefly, CD34+ cells were thawed, and 100,000 cells were seeded into a 96-well round-bottom plate in 200 µL of media. Half the media was removed and replaced with fresh media, plus the lentivirus, the following day. Cells were transduced at an MOI of 50. The following day, cells were counted, and 100,000 live cells were moved to a 6-well Grex for the remaining 19 days of expansion with media changes every 3–4 days. Cells were then stored as described above for unmodified cells.

### 2.3 Preparing cells for injection

All work was done aseptically. Cells were thawed quickly, resuspended in 15 mL sterile RPMI with 1% FBS, and then centrifuged, followed by resuspension in 110 µL cold RPMI with 1% FBS. 10 µL of cell suspension was used to count the cells and determine viability. Cells were then diluted to 200 µL with cold RPMI with 1% FBS and transferred to a microcentrifuge tube for transfer to the animal facility on ice. Once at the animal facility, the cells were kept on ice until a fetus was located by ultrasound and was deemed viable for injection. When a fetus was ready for injection, the cells were lightly flicked to mix before being drawn up with a sterile 23-gauge needle and a 1 mL syringe.

### 2.4 Fetal liver injection surgeries

Sows for fetal liver injection were maintained on 15 mg 0.22% Altronogest via oral administration of 6.8 mL Matrix (Merck), once daily from arrival in the facility until the day before the c-section. Sows were 41–42 days gestation on the day of fetal liver injection. Sows were administered with acepromazine (0.2 mg/kg) intramuscularly 60–90 minutes prior to transportation to the surgery preparation room. In the preparation room, sows received 0.175-0.185 mg/kg morphine sulfate intramuscular and topical lidocaine cream on the ears. After approximately 10–15 minutes of contact time for the lidocaine, an ear catheter was placed, and propofol was administered IV to effect (target was 600 mg) (18). Once a sufficient anesthetic plane was achieved, the sow was intubated, and anesthesia was maintained on isoflurane administered with oxygen. The surgical site was shaved, and an initial scrub was performed in the preparation room.

Each sow was moved to the surgery room and placed in dorsal recumbency on the surgery table. The sow was maintained on IV fluids of Lactated Ringer’s Solution +/- 5% dextrose for the duration of the surgery (sows received a total of 3 liters each). Heat support was provided with a forced-air warming device. A complete surgical scrub was performed. Surgery was done using a standard sterile technique. A ventral midline incision was made, and the uterus was exteriorized. An ultrasound was performed of the entire uterus to identify all fetuses; fetuses were marked on the uterine wall using a sterile regular-tip surgical skin marker (Medline). The fetuses to be injected were chosen, the liver was identified on ultrasound, and the injection was placed intrahepatic/intraperitoneal. The injected fetuses were identified by stitches placed in the uterine wall with 0 polypropylene monofilament (Securopro) suture (19, 20). Once all fetal injections were complete, the uterus was lavaged, replaced in the abdomen, and the abdomen was lavaged with up to 1 liter of warmed sterile saline. The abdomen was closed in 3 layers: the abdominal wall and subcutaneous layer using 0 Vicryl and the skin using 1 PDS in a ford interlocking pattern. The skin surrounding the incision was cleaned with Sureprep, and the incision was covered with Tegaderm. Each sow was administered Meloxicam (0.3 mg/kg) intramuscularly, Cefazolin (25 mg/kg) intravenously, and Buprenorphine ER (Wedgewood/Zoopharm) intramuscularly (0.18 mg/kg) during surgery. Animals were monitored post-surgery until awake and alert; recovery was uneventful. The Tegaderm bandage on one animal came off in recovery. Animals were monitored at least twice daily in the post-surgical recovery period and dosed with meloxicam (0.3 mg/kg per os) once daily for a minimum of 2 days post-surgery. Sutures were removed ~2 weeks post-surgery. No sows required antibiotics and no sows aborted a pregnancy after fetal liver injection. We replicated the expansion of cells for fetal liver injection surgeries three times and used these cells across five different litters of SCID pigs.

### 2.5 Cesarean section to obtain piglets

After fetal liver injection surgery, sows were carefully monitored to ensure pregnancy maintenance until day 119 of gestation. On day 119 of gestation sows were sedated with 0.15-0.2 mg/kg intramuscular acepromazine (MWI Animal Health, Boise, ID) in the housing room for 40–60 minutes before moving to the surgery preparation room. A similar sedation processed was followed as was used for the fetal liver injection surgery. Once properly positioned, a complete surgical scrub was performed. Spray-on adhesive was used to secure a homemade flexible film surgical bubble to the sow. The surgical bubble and contents had been previously autoclaved where possible (surgical instruments and metal transfer cylinder) and sterilized with Clidox (Pharmacal Research Laboratories, Waterbury, CT) spray (1:3:1). The surgical bubble allowed gloved access for the surgeon and surgery assistant as well as four people to revive piglets. A paramedian incision was made into the abdominal cavity, and the uterus was exteriorized. Once the uterine horn was exposed at c-section, each piglet was recovered according to their injected position and identification suture on the uterine wall of the uterine horn to identify the cell injection treatment the piglet received previously. Incisions were made over each piglet; as each piglet was removed, the umbilicus was clamped, and 1–2 drops of naloxone (Somerset Therapeutics LLC, Hollywood, FL) were placed on the tongue. Piglets were stimulated to assist with revival and warmed with towels and hand warmers. Any piglet that was still sedated approximately 10 minutes after removal from the uterus had 1–2 additional drops of naloxone administered. The sow was euthanized after all piglets had been removed. Of the 15 fetuses that were injected, three did not survive. All of these three were injected with experimental CAR-T genetically modified cells; thus, we cannot verify that these modified cells are viable in an *in vivo* setting. These injections were part of a pilot study included with the other CD34+ HSCs.

### 2.6 Blood and tissue collections

Blood samples were taken intravenously into Vacutainer EDTA tubes and processed within 6–18 hours for flow cytometry (see below). A standard necropsy was performed by a veterinarian on every SCID pig that died or was euthanized, including samples taken during every necropsy to evaluate each pig for signs of humanization. The following samples were taken and fixed in 10% formalin for histology and immunohistochemistry: tonsil, heart, lung, thymus, liver, kidney, spleen, testicle or ovary, mesenteric lymph node, peripheral lymph node, duodenum, jejunum, ileum, spiral colon, cecum, bone marrow, adrenal gland, pancreas. The following tissue samples were also collected in media: tonsil, lung, thymus, liver, spleen, mesenteric and peripheral lymph node, spiral colon, and adrenal gland for cell dissociation and flow cytometry. Only SCID pigs that were later found to be humanized routinely had gross evidence of a thymus and mesenteric or peripheral lymph nodes.

### 2.7 Tissue preparation and flow cytometry

Tissues were kept in media on ice until ready for processing. For each tissue sample, tubes were prepared with a 70 µm cell strainer (Falcon). Tissues were minced in a petri dish with 5 mL of media using a scalpel and then passed through the cell strainer. Using a 5 mL syringe plunger, the tissue mixture was pushed through the strainer, adding media to wash it as needed, until the final sample volume reached 10 mL.

For whole blood samples and for tissue samples after processing, samples were centrifuged (1700 x g for 6 minutes) at room temperature. The supernatant was removed without disturbing the pellet. The pellet was resuspended in 3–4 mL of 1X red cell lysis buffer (Abcam, 10X RBC Lysis, 50-221-5374) and incubated for 5–10 minutes until the solution cleared. An equal volume of PBS with 1% FBS was added, mixed, and the sample was centrifuged again to remove the lysis buffer. The supernatant was discarded, and the pellet was washed with PBS containing 1% FBS. The samples were washed once more with PBS/1% FBS before centrifuging again and decanting. Compensation beads were vortexed, and one drop was added to each compensation tube. After the final spin, the supernatant was removed, and the sample was transferred to the appropriate flow tube. Antibodies were prepared in a master mix and added to each sample. A list of antibodies used for the human subsets is provided in **Table 3**.

**Table 3:** Flow cytometry antibodies used for assessing human cell subsets.

Fluorochrome	Target protein	Amount/assay	Clone/Catalog
eFluor 780	Fix Viability	1 µl	Invitrogen,65-0865-14
FITC	pCD45	5 µl	Bio-Rad, MCA1222F
BV785	hCD45	5 µl	BioLegend,304048
PE	hCD3	2 µl	BioLegend,300408
Alexa Fluor 700	hCD4	5 µl	BioLegend,300526
BV605	hCD8α	5 µl	BioLegend,301040
BV421	hCD19	5 µl	BioLegend,302234
PE-Cy7	hCD56	5 µl	BioLegend,318318
APC	hCD163	5 µl	BioLegend,333610
AF700	hCD34	1uL	BioLegend,343622
PE	hCD38	1uL	Biolegend,303506
APC	hCD45	1uL	BioLegend,304021
BV605	hCD45Ra	1uL	BioLegend,304134
BV421	hCD90	1uL	BioLegend,328122

Antibodies were added to the samples and then incubated (4 °C for 30 minutes). After 30 minutes, samples were washed with 1 mL of PBS with 1% FBS. Spin the sample, discard the supernatant and decant the sample. Add 2% formaldehyde to fix the samples and incubate (4 °C for 20 minutes). Samples were then rewashed and passed through a filter (Flowmi Cell Strainers, BAH136800040) into new flow tubes before undergoing flow cytometry analysis to ensure they were free of clumps.

FlowJo v.10 (Becton, Dickinson & Company) was used to analyze the results for the human subsets. The process begins with creating a gate on a forward scatter (FSC) versus a side scatter (SSC) plot. This shows the populations of cells based on size (FSC) and granularity (SSC). This initial gating also separates viable cells from debris. Doublets are excluded by using FSC-A (area) versus FSC-H (height). Cells that fell on the diagonal of the area versus height plot are the single cells that were used for further human leukocyte gating.

### 2.8 Histology

Lymphoid tissues from SCID pigs engrafted with human cells were analyzed with routine histology and immunohistochemistry, as previously reported (Adeline N. 11). Tissues were immunolabeled for anti-CD3ϵ (Dako #A0452) and anti-Pax5 (Ventana clone 24) to assess for the presence of human T and B cells, respectively. Briefly, CD3ϵ labeling deparaffinization, antigen retrieval, immunohistochemical labeling, and counterstaining were performed on the BOND-MAX automated staining system (Leica BioSystems, Buffalo Grove, Illinois) using a diaminobenzidine (DAB) detection system (Leica BioSystems) with standard ER1 retrieval for 20 minutes. For PAXx5, deparaffinization, antigen retrieval, immunohistochemical labeling, and counterstaining were performed on the Discovery Ultra automated staining system (Ventana Medical Systems, Tucson, Arizona) using an alkaline phosphatase ultra-red detection system (Ventana Medical Systems) with standard CC1 antigen retrieval for 64 min.

## 3 Results

### 3.1 Expansion of cells increases opportunity for humanization in cloned RG SCID pigs

Successful prenatal humanization of DCLR1EC-/-IL2RG-/Y SCID pigs, using approximately 1–2 million expanded CD34+ cells, has been reported (11), but the number of T and B cells found in tissues were low (T) and very low (B). We used new protocols to expand umbilical cord blood cells 100–200 fold (**Supplementary Figure 1**; **Supplementary Table 1**) without loss of predicted fraction of hHSC (CD34+CD45+CD38-CD45Ra-CD90+). Once pregnancy was confirmed at day 35 of gestation, the pregnant females were shipped to ISU to acclimate before the laparotomy to be performed on day 41–42 of gestation. Confirmed hCD34+ expanded cells were thawed and prepared in media for each injection the morning of the surgeries. As these litters are expected to be 100% SCID, some of the fetuses were injected, and some of the fetuses were left noninjected as controls, as well as to decrease the chances that the litter would be aborted due to fetal mortality. Pregnancy viability was high post-injections, and remained the same as other SCID litters that did not receive injections (100% of litters used for *in utero* humanization attempt, as well as those not injected, carried the pregnancy to term). All injection attempts across all litters are listed below (**Table 4**).

**Table 4:** Summary of injection attempts of SCID pigs across different litters.

Pig ID	Injected/noninjected	# Cells injected	Gestation day injected
18-1	injected	20 million	41
18-2^	injected	CAR-T 20 million*	41
18-3^	injected	CAR-T 40 million*	41
18-4^	noninjected	–	–
18-5^	noninjected	–	–
18-6	noninjected	–	–
18-7^	noninjected	–	–
18-8	noninjected	–	–
18-9^	noninjected	–	–
18-10^	noninjected	–	–
18-11^	noninjected	–	–
19-1^	injected	40 million	41
19-2	injected	40 million	41
19–3 ^	injected	CAR-T 20 million*	41
27-1	injected	20 million	41
27-2	noninjected	–	41
27-3	injected	20 million	41
27-4	noninjected	–	41
27-5	injected	20 million	41
27-6	injected	40 million	41
27-7	injected	40 million	41
30-1	injected	20 million	42
30-2	noninjected	–	42
30-3	injected	20 million	42
30-4	noninjected	–	42
30-5	noninjected	–	42
31-1	injected	20 million	42
31-2	noninjected	–	42
31-3	noninjected	–	42
31-4	injected	20 million	42
31-5	noninjected	–	42

All fetuses that were present during a fetal liver injection are listed, along with injection status, the number of cells injected, and the day of gestation the fetal liver injection surgery took place. CAR-T, chimeric antigen receptors-T cells. *A genetically modified cell type intended to express a chimeric antigen receptor (data not shown); no animals injected with these cells were found at necropsy. ^Indicates that the animal did not have a flow cytometry analysis performed.

### 3.2 Improved humanization of SCID pigs injected with 20 million or greater expanded cells

**Figure 1:**
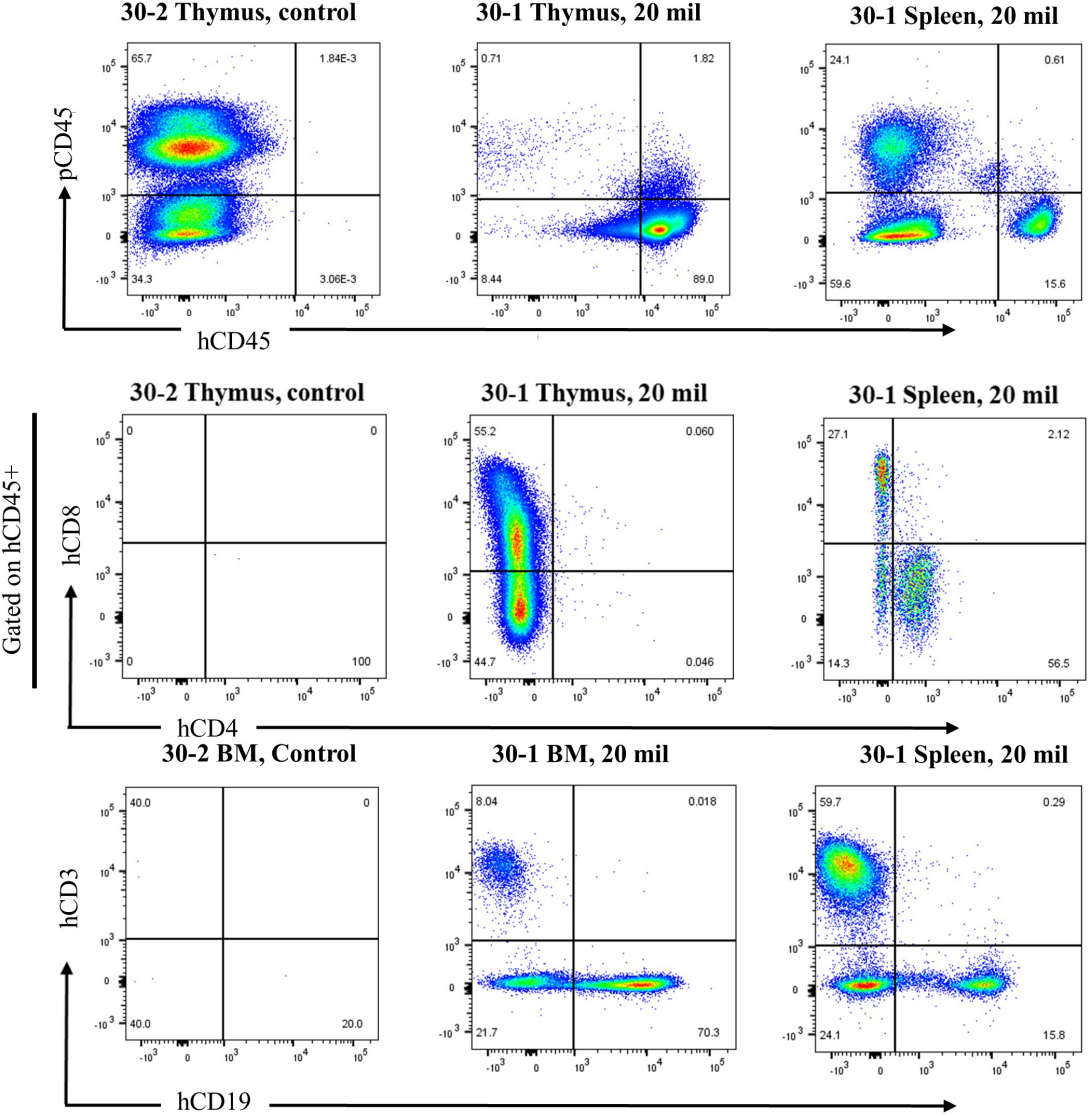
SCID pigs injected with 20 million CD34+ cells have significant humanization. Lymphocytes from thymic tissue are identified by morphology, then separated into porcine CD45 and human CD45 populations, and then into CD3 and CD19 subsets. Those CD3+ cells were further gated into CD4 and CD8 T cells. The left column shows a control animal that has not been injected, showing no cells positive for human CD45 or other subsets. Each of the right two columns shows an animal injected with 20 million expanded CD34+ cells with a significant population of hCD45+/hCD3+ (T cells) and hCD45+/hCD19+ (B cells).

Once all piglets were identified, most piglets were euthanized approximately 30 minutes after birth, after blood collection, and tissues were collected to document the level of humanization and minimize the potential effects of the resident innate immune system on human cell survival. Litters 30 and 31 (listed in tables) were euthanized after 1 week to collect tissues. None of the fetuses injected with the CAR-T modified cells survived to term, and all remaining data discussed transplantation using un-modified human cells. Relevant tissues, including thymus, spleen, liver, lung, and BM as well as whole blood, were processed for histology and flow cytometry. In flow cytometry, we used markers that specifically identified both porcine and human immune cells (pCD45 and hCD45, respectively) and markers for specific human leukocyte subtypes. An example data set showing results for two tissues from an injected and a non-injected pig are shown in **Figure 1** (thymus and spleen). As expected, there is a lack of human cells detected in the non-injected control pigs. The hCD45+ cells detected in a humanized SCID pig were then gated for other markers to determine sub-cell types, including CD3+ (T cells), CD19+ (B cells), and CD163+ (myeloid) cells. Further gating subtyped the CD3+ cells into CD4+ (T helper cells), CD8+ (T cytotoxicity cells), and CD4+CD8+ (double-positive T cells) (**Figure 1**). In the injected pigs, flow cytometry revealed that the number of human lymphocytes in tissues was significantly higher than in prior published work, which used 1–2 million CD34+ cells per prenatal injection with human CD45+ levels of 53-58% in the thymus of two animals (11). There was a significant population of T (hCD45+CD3+) and B (hCD45+CD19+) cells in the thymus of this example animal (**Figure 1**), which is #30–3 from **Tables 4** and **5**. The number of hCD45+ CD163+ cells detected was minimal; however, these animals have not received exogenous T cell stimulation, which would activate cytokine production in the myeloid cells (21, 22).

**Table 5:** Results for flow cytometric analysis of thymus of liveborn SCID piglets.

Pig ID	Category of humanization	Total human in all CD45+ leukocytes(%)	%CD3+ (T)	%CD+ CD4+ (T helper)	%CD3+ CD8+ (T cytotoxic)	%CD3+ CD4+ CD8+ (Double Positive)	%CD19+ (B)	%CD163+ (Myeloid)
18-1	Humanized	136440 (81.06)	66.70	58.60	29.20	2.67	1.70	0.63
19-2	Humanized	186800 (80)	61	37.50	40.30	11.30	1.20	0
27-1	Humanized	2503 (7.31)	16	0	4	0	0	21
27-3	Humanized	109804 (85.7)	57.50	6.05	38.30	52.10	0	52.30
30-1	Humanized	144768 (89)	60.70	55.40	44.70	2.51	25	6.25
30-3	Humanized	36784 (19)	18.80	84.40	8.42	1.60	43.40	7.97
27-5	Very low	85 (1)	1.22	0	66.70	0	6.50	92.30
27-6	Very low	60 (1)	0	0	0	0	0	0
27-7	Very low	305 (2.50)	60.70	52.40	32.30	8.35	0.83	37.90
31-1	Very low	31 (0.35)	0	0	0	0	69.40	28.60
31-4	Very low	23 (0.32)	0	0	0	0	16.20	33.30
19-1	Injected, not humanized	0 (0)	0	0	0	0	0	0

In the column for total human CD45+ cells, the total such cells detected is provided, followed by the percentage of total CD45+ cells in parentheses. In each succeeding column, the number shown is the % of cells positive for the human marker protein or protein combination shown. Predicted cell type is shown in parentheses in headers.

**Figure 2:**
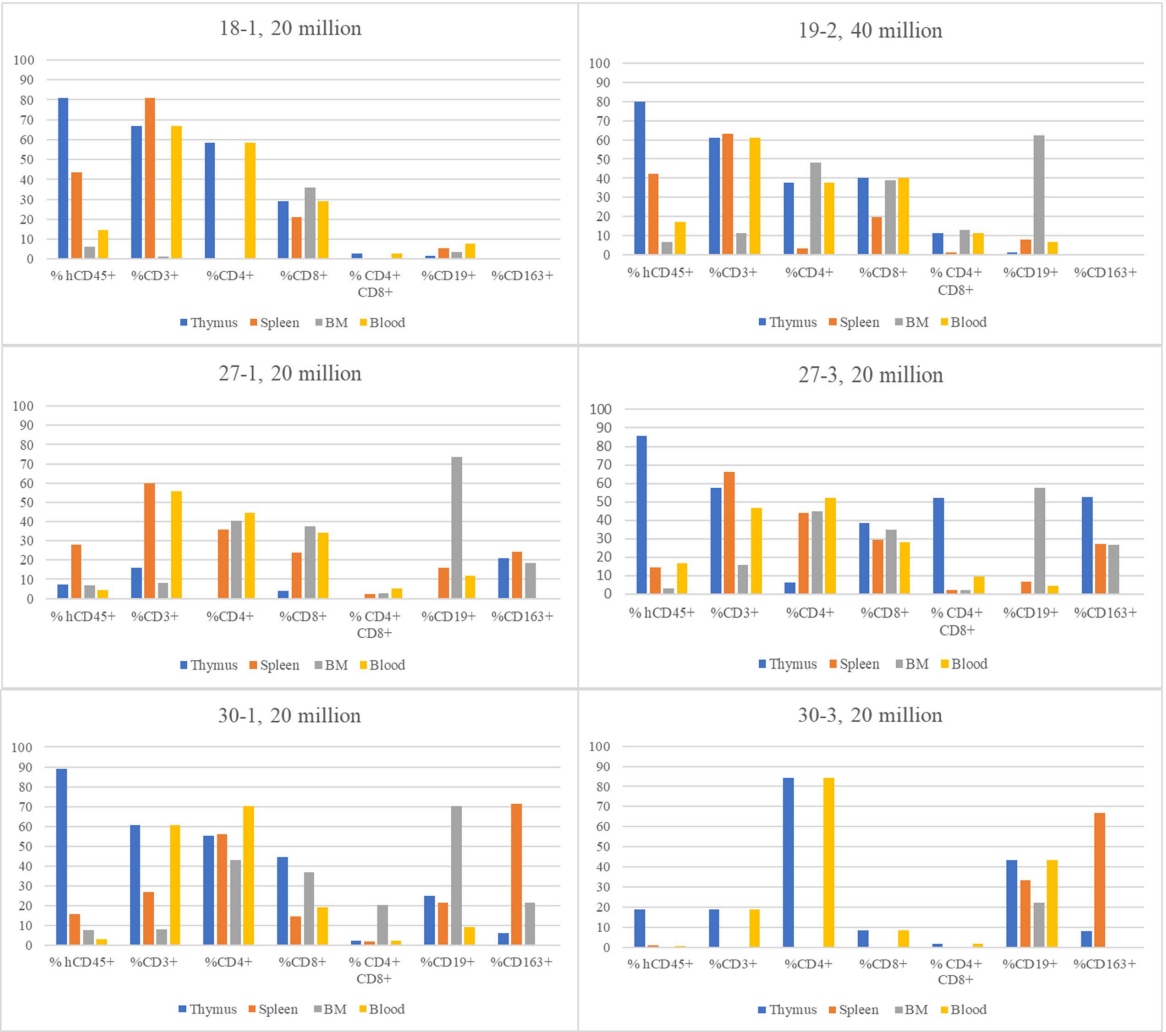
Comparison of flow cytometric results across all tissues for six animals with highest % of total human CD45+ cells. Graphs show the % positive of human lymphocytes that were gated for markers shown in thymus, spleen, bone marrow, and blood samples. The first set of columns are shown as a percentage of all mononuclear leukocytes. All other data is provided as a percentage of these human CD45+ cells identified.

Summary results for flow cytometry analysis for blood and three tissues collected from all SCID live-born neonatal pigs derived from fetal injections are shown in **Tables 5**, **6** and **Supplementary Tables 2** and **3**. Cells from tissues from the eleven non-injected pigs were analyzed by flow cytometry, and did not show any human CD45+ cells (data not shown). One exception to this lack of detected human lymphocytes was pig #30-2, which had 0.045% hCD45+ cells that did not stain for any T, B, or myeloid lineage markers. A graph depicting the percentage of various human cell types across all tissues for six injected pigs with the highest levels of human CD45+ cells is shown in **Figure 2**, to provide a direct comparison across immune tissues within animal, as well as a comparison to the other humanized pigs. It is not possible to determine with the existing data whether 20 versus 40 million cells injected affects the rate of humanization, as the number of direct comparisons between 20 and 40 million was insufficient for a statistical test. However, the data does support the conclusion that 2–5 million used by Boettcher et al. is insufficient. We expect the variation across attempts to be due to both the number of cells successfully transferred into the fetus and the location of the injected cells.

**Table 6:** Results for flow cytometric analysis of blood of liveborn SCID piglets.

Pig ID	Category of humanization	Total human in all CD45+ leukocytes (%)	%CD3+ (T)	%CD+ CD4+	%CD3+ CD8+	%CD3+ CD4+ CD8+ (Double Positive)	%CD19+ (B)	%CD163+ (Myeloid)
				(T helper)	(T cytotoxic)			
18-1	Humanized	3085 (14.52)	66.70	58.60	29.15	2.70	7.60	0
19-2	Humanized	3683 (17.20)	61.01	37.47	40.23	11.30	6.65	0
27-1	Humanized	15913 (4.02)	55.90	44.60	34.10	5.14	12	0
27-3	Humanized	19302 (16.70)	46.80	52	27.90	9.35	4.37	0
30-1	Humanized	2039 (3.28)	60.70	70.40	19.40	2.51	9.17	0
30-3	Very low	2659 (0.76)	18.80	84.40	8.42	1.60	43.40	0
27-5	Very low	246 (0.078)	1.22	33.30	66.70	0	6.50	0
27-6	Very low	0 (0)	0	0	0	0	0	0
27-7	Very low	2287 (0.74)	60.70	52.40	32.30	8.35	0.83	0
31-1	Very low	49 (0.03)	0	0	0	0	69.40	0
31-4	Very low	31 (0.01)	0	0	0	0	83.80	0
19-1	Injected, not humanized	0 (0)	0	0	0	0	0	0

In the column for total human CD45+ cells, the total such cells detected is provided, followed by the percentage of total CD45+ cells in parentheses. In each succeeding column, the number shown is the % of cells positive for the human marker protein or protein combination shown. Predicted cell type is shown in parentheses in headers.

### 3.3 Fetal stage humanization provides consistent thymic humanization

Of the 12 fetuses injected with unmodified human CD34+ cells, 11 of the liveborn pigs were found to have at least minor levels of human leukocytes present in their thymus tissue via flow cytometry analysis. We identified two categories of such humanization. The first, considered humanized, had leukocyte populations in the thymus comprised of > 5% human CD45+ cells (**Table 5**). The second category, termed very low, clearly had many fewer human cells (from 0.32 to 2.5% of the total). In the >5% humanized group, the tissues have relatively high CD3+ T cells, CD4+, and CD8+ subsets (**Table 5**), but the CD19 and CD163 are low or zero for many humanized samples. Samples with very small amounts of human cells on flow seem to have higher CD19 and CD163, but very few or no CD3 cells.

Overall, the fractional CD45+ population in the spleen was lower than observed in the thymus of the same animal (**Supplementary Table 2**). Using the same categorization as for the thymus, four animals can be categorized as having spleens with human engraftment, and six animals with very low human engraftment. All pigs except pig #30–3 in the humanized thymus group were also humanized in the spleen. The group with > 14% CD45+ human leukocytes in the spleen has a higher overall fraction of T cells than B cells. For the category of very low human leukocytes, most of these animals have a higher fraction of B cells compared to T cells. In both groups of pigs, some pigs showed a significant population of myeloid cells, with the very low group having a higher incidence of detectable myeloid cells compared to the more humanized group.

Among animals with the highest level of humanization in thymus tissue, the flow results of BM tissue showed a consistent amount of CD45+ human cells in the humanized pigs to be around 6-7% of all cells analyzed (**Figure 2**; **Supplementary Table 3**). Within the subsets of the CD45+ human cells found in the bone marrow, a majority (50-80%) of the cells were categorized as CD19+ (B cells) and a sizeable (18-45%) portion as CD163+ (myeloid) cells. The analysis of the blood showed that only 5 of the animals with a proportion of human cells could be considered engrafted (3-17%), with a higher proportion of CD3+ cells than CD19+ cells (**Table 6**).

**Figure 3:**
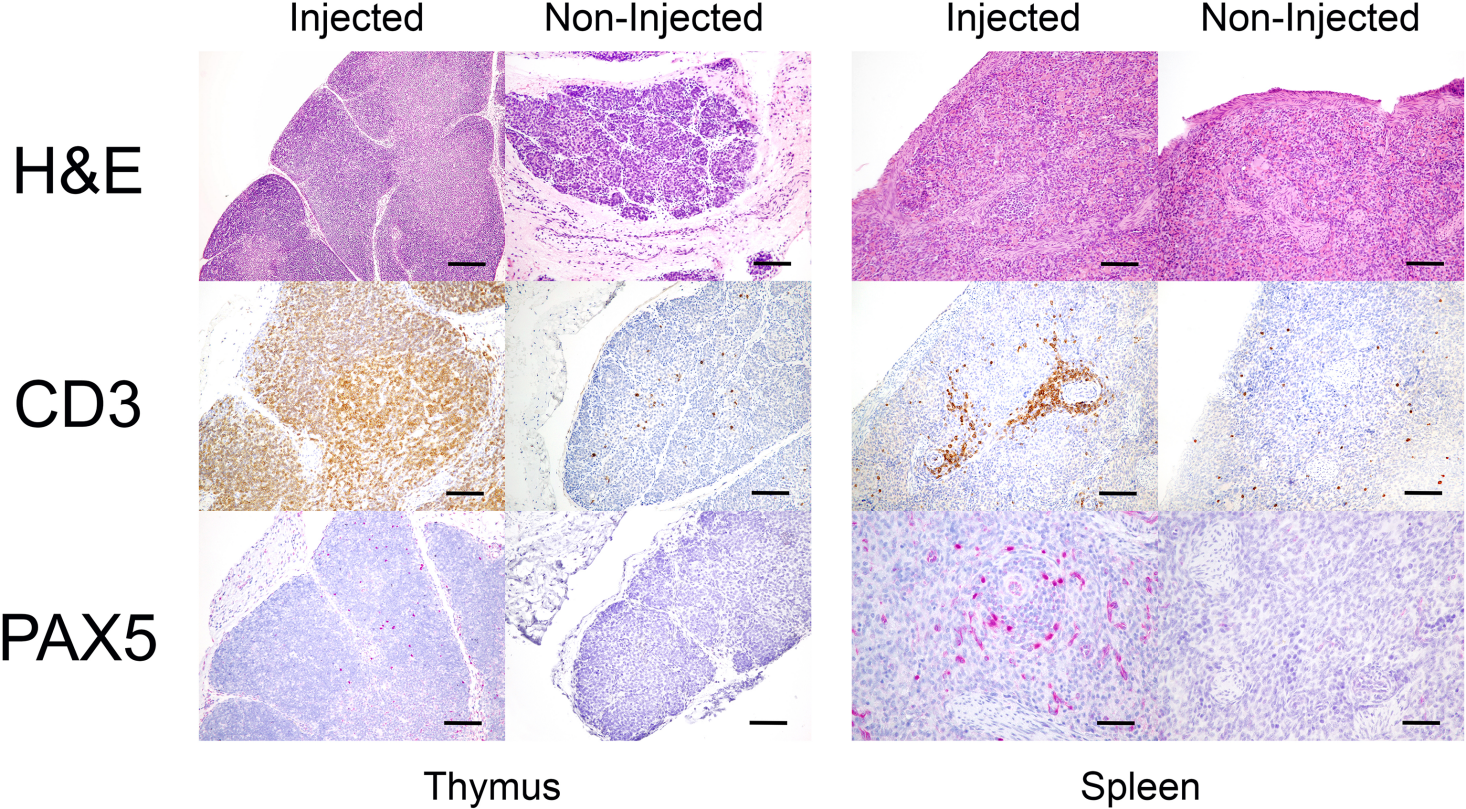
Human cells present in the lymphoid organs of injected SCID pig. Normal thymic histology of the SCID pig injected with 20 million expanded CD34 +. The thymus section of an injected SCID pig (30-3) is on the left side, presented for H&E, T cell labeling, and B cell labeling (top to bottom). The thymus section of a SCID pig that was not injected (30-2) is on the right, with the same immunolabeling. Spleen from injected (30-3) and non-injected (30-2) on the right shows CD3 cells present in periarteriolar sheaths. PAX5 cells are dispersed through the injected tissue, with the structure more pronounced than in the noninjected pig on the far right. Note the small number of CD3+ cells labeled in the non-injected pig section for both thymus and spleen. Scale bar, 150 µm, except for PAX5 panel of the spleen for the non-injected animal, is 100 µm.

Histological structures of thymus and spleen tissues were also examined in SCID pigs injected with human CD34 cells or not injected. Although a hallmark of SCID physiology is the poor development of the thymus due to a lack of thymocyte precursor differentiation, thymic structure and human cell content was substantial in several injected animals. **Figure 3** shows an example from a pig that was injected with 20 million cells (#30-3), which had 18.8% of the hCD45+ cells that were also positive for CD3+ (**Table 5**). Thymus from this pig showed large lobules composed of medulla and cortex, with densely packed small T cells. At the same time, thymic tissue is markedly hypoplastic in non-injected SCID pigs as evidenced by small lobules that are depleted of lymphocytes and have no distinguishable medulla and cortex (**Figure 3**). Similarly, within the spleen of humanized pigs, periarteriolar lymphoid sheaths (PALS) are primarily populated by T cells and surround central arteries, as well as small aggregates of B cells resembling early primary follicles. No significant development of spleen structure was observed in noninjected pigs (**Figure 3**). While a few scattered CD3+ cells were detected in both thymus and spleen of 1 non-injected pig, rare immunopositive cells represent non-specific binding, as confirmed by flow cytometry of the same tissue samples did not detect either human or pig CD3+ cells in any non-injected pig. Bone marrow of injected animals was analyzed, but due to the size of the sample required for decalcification and fixation, distinction of definitive hematopoietic cell lineage was not possible. These analyses demonstrate that pre-natal administration of 20 million or more hHSCs into RAG2-/-; IL2RG-/Y pigs results in extensive human cell differentiation in multiple tissues.

## 4 Discussion

This study presents significant advancements in the development of a humanized SCID pig model using an optimized prenatal cell transplantation approach, achieved by injecting 20–40 million expanded human CD34+ cord blood cells into RG SCID fetuses at 41–42 days of gestation, which is during key hematopoietic development in liver and bone marrow (23). This timing is also where fetuses are able to accept and develop exogenously injected cells (24). Using flow cytometry and histological analysis, we observed engraftment and differentiation of human immune cells in 11 of 12 live-born pigs, with more than half demonstrating substantial levels of humanization (>5% hCD45+ cells). We conclude that this approach to introducing human cells as early as possible during normal pig hematopoietic ontogeny may be beneficial for maximizing human hematopoiesis and for effective interaction with pig thymic tissue during prenatal immune system maturation.

Our data show that this approach markedly improves lymphoid reconstitution compared to earlier attempts using lower cell doses (8, 11). In the thymus, human T cells (hCD45+CD3+) were the predominant population, with a range of helper (CD4+), cytotoxic (CD8+), and double-positive (CD4+CD8+) subsets present. In contrast, B cells (CD19+) and myeloid cells (CD163+) were present, albeit in lower frequencies—especially in pigs with higher overall T cell chimerism. These findings are consistent with the expected developmental trajectory of the immune system and align with prior reports in large-animal models, which have noted difficulty in achieving robust B and myeloid lineage reconstitution using human CD34+ cells alone (8, 11, 24). Our prenatal injection approach also results in substantial thymic tissue restoration, with histological structures similar to the results found by Hu and colleagues in postnatal injections only with their triple knockout line (12).

Spleen and bone marrow analysis added further depth to our assessment of the impact of human immune cell differentiation on SCID immune organ development. Substantial hCD45+ cell populations, predominantly T cells, were observed in the spleen with variable but detectable populations of B cells and myeloid cells. Interestingly, in animals with only minimal thymic humanization, the spleen contained a relatively higher proportion of B cells and CD163+ myeloid cells, suggesting these lineages may engraft or persist under different conditions or at different kinetics than T cells. The CD45+ human cells found in blood in our RG model are slightly lower (6-7%) than that of the Hu et al. RG model (11%), in which the animals were post-natally injected in the BM with CD34+ human cells (12). The spleen in our RG cohort shows a different outcome with higher engraftment of human CD45+ cells, 15-43%, as compared to <1% reported by Hu et al. or < 11% as reported by Sper et al.

Histologically, the thymus of the prenatal humanized pigs displayed well-defined cortical and medullary regions, a hallmark of normal thymic architecture, which is typically absent in untreated SCID pigs. The presence of densely packed CD3+ T cells and scattered Pax5+ B cells supports the involvement of human hematopoietic cells with the maturing pig thymic tissue and suggests that human T cell development can proceed within the porcine thymic microenvironment. Hu and co-workers reported improved thymic development in their humanized RGD model compared with their RG model. Interestingly, we have seen similar increased hallmarks of thymic development, including full-sized lobules with little connective tissue between the lobules and a well-developed cortex and medulla, in our RAG1-/- IL2RG-/Y model. This may reflect that the human cells are present in the porcine thymus much earlier in our procedure, potentially providing important signals and/or interactions with porcine thymic epithelial cells that only occur much later in the Hu postnatal procedures. It would be of interest to test whether the level of humanization in the RGD model can be further improved using the fetal administration approach.

Similarly, spleens from humanized *RAG2-/-; IL2RG-/Y* pigs showed improved structural organization, including periarteriolar lymphoid sheaths (PALS) populated by CD3+ cells and small lymphoid aggregates, which were absent in non-injected controls. These features are also indicative of early lymphoid organ development and immune compartmentalization. Bone marrow from prenatal humanized pigs contained representatives of all major hematopoietic lineages by flow cytometry, although the inability to perform immunohistochemistry due to necessary decalcification of the bone highlights the technical challenges of evaluating BM engraftment at this early stage.

This work supports the hypothesis that increasing both the dose and timing of CD34+ cell injection enhances engraftment and thymopoiesis in SCID pigs. We summarize in **Table 7** several methods that have been reported to introduce human hematopoietic stem cells, with varying degrees of human immune cell development. This table highlights that the approach detailed in our methods for *in utero* implantation in the RG model achieves a higher proportion of substantially humanized pigs at birth than earlier fetal approaches, with 11 of 12 liveborn animals engrafted and over half reaching at least 5% hCD45 cells in key lymphoid tissues. These results also show a higher number of human cells in tissues compared to a RG postnatal implantation model (12). The overall current best model for humanization is the RGD model (12), but comparison of our current results with the RG model of Hu et al. could indicate that *in utero* injection into RGD genotype pig models may further improve humanization in SCID pigs.

**Table 7:** A comparison of SCID pig models and humanization levels achieved.

	Boettcher et al.. 2019 (11)	Sper et al. 2022 (8)	Hu et al. 2025 (12)	(Forster et al. (this study))	Hu et al. 2025 (12)
Breed	Yorkshire	Yorkshire x Landrace x Duroc crossbreed	Bama (mini)	Yorkshire and white crossbred	Bama (mini)
Genetics	Natural Artemis mutation plus IL1RG	IL2RG-, RAG2-, (RG)	IL2RG-, RAG2-, (RG)	IL2RG-, RAG2- (RG)	IL2RG-, RAG2-, CD47- (RGD)
Conditioning	None	None	Busulfan IP	None	Busulfan IP
Cells used	Expanded umbilical cord blood	Mobilized PBMCs	Fetal liver CD34+	Expanded umbilical cord blood	Fetal liver CD34+
Number of Cells	2–4 million	7 million	6–16 million	20 (n=10) or 40 million (n=1)	6–16 million
Injection method	*In utero*, intraperitoneal/liver	*In utero*, intraperitoneal/liver	Postnatal, intraosseous	*In utero*, intraperitoneal/liver	Postnatal, intraosseous
Results	2/2 fetuses some hCD45+, 2/2 showing 1.6-21% in cord blood, 0.02-0.2% in Bone marrow, 3.8-6% in spleen and with 50-60% in thymus	4/7 some hCD45+; 4/7 showing 3-4% in cord blood; with example humanized pig tested for day 1 bone marrow (0.7%) spleen (10%), and thymus (detected)	6/6 some hCD45+; 2/4 with 0.45-2.5% in blood; 6/6 showing 2-45% in bone marrow; 5/6 with 0.04-0.43% in spleen;	11/12 some hCD45+; 5/11 with 3-17% in blood; 6/11 showing 0.2 to 7% in bone marrow, 5/11 with 15-43% in spleen; 6/11 with 7.3-89% in thymus	9/9 some hCD45+; 9/9 showing 12-90% in blood; 3/9 reported 55-95% in bone marrow; 3/9 reported 5-75% in spleen

Variations in engraftment success suggests that this *in utero* approach could be optimized for improved humanization. It is important to note that differences in engraftment could be due to the exact injection of CD34+ cells being either intrahepatic or intraperitoneal, with the goal being intrahepatic. Our method of *in utero* injection of CD34+ expanded human cells (19) from umbilical cord blood avoids the use of fetal liver tissue, as well as any preconditioning such as total body irradiation, yet demonstrates that substantial numbers and types of human immune cells are established at birth. The successful use of *in vitro* expanded hHSC from umbilical cord blood for SCID pig humanization also extends these methods beyond those groups with facilities required for purification of CD34+ cells from mobilized peripheral blood. This study was limited to flow cytometric analyses of these human cells, and future studies will focus on measuring human cell differentiation beyond birth to test for further maturation of immune structures and function, including responses to mitogenic stimuli and evaluation of antigen-specific immunity. In using our methods for a conventional sized pig (larger breed size, faster weight gain), coupled with the CD47 deletion, the longevity of human immune cell subtypes could be further studied to develop a model that better recapitulates human immune system and biomedical model.

In conclusion, the methods described here adds approaches to improving the humanized SCID pig model, which holds strong promise as a translational platform for preclinical research. With continued refinement, the humanized SCID pig model may provide a physiologically relevant system for studying human hematopoiesis, immune responses, and therapeutic interventions in ways that are not possible with current rodent models.

## Data Availability

The original contributions presented in the study are included in the article/**Supplementary Material**. Further inquiries can be directed to the corresponding authors.

## Supplementary material

Supplementary Figure 1 UCB-CD34+ cell phenotype before and after expansion. Human-derived CD34+ cells were pre-gated on CD45+ viable cells and evaluated for progenitor and stem cell phenotype.

Supplementary Table 1 Calculations for UCB-CD34+ progenitor and stem cell phenotype used.

Supplementary Table 2 Results for flow cytometric analysis of spleen of liveborn SCID piglets.

Supplementary Table 3 Results for flow cytometric analysis of bone marrow of liveborn SCID piglets.
