# Supplementary material for: Midgestational injection of highly expanded human CD34+ cells increases lineages of human immune cells and supports thymic development in RAG2-/-IL2RG-/Y SCID pigs
Source: Front Immunol. 2026 Mar 11;17:1751541. doi: 10.3389/fimmu.2026.1751541 (PMC13012948; doi:10.3389/fimmu.2026.1751541)
Supplement: Supplementary Table 1 — Calculations for UCB-CD34+ progenitor and stem cell phenotype used. [file DataSheet1.docx]

# Supplementary Tables

Supplementary Table 1: Calculations for UCB-CD34+ progenitor and stem cell phenotype used

| **Sample** | **CD45%** | **CD45+**  **CD34+ CD38-** | | **CD45+CD45Ra-CD90+** | **Total HSC (CD45+CD34+CD90+CD38-CD45Ra-)** | | **% HSC (CD45+CD34+CD90+CD38-CD45Ra-)** | **Total Amount of Cells** | | **Total Amount of Cells with HSC phenotype** |
| --- | --- | --- | --- | --- | --- | --- | --- | --- | --- | --- |
| UCB Day 0 | 1.0000 | 0.0119 | | 0.1418 | 0.0017 | | 0.1687 | 1000000 | | 1687 |
| UCB Day 0 | 0.8600 | 0.0428 | | 0.0415 | 0.0015 | | 0.1528 | 1000000 | | 1528 |
| Expansion 1  No LV Day 21 | 1.0000 | 0.224 | | 0.0038 | 0.0009 | | 0.0851 | 110000000 | | 93632 |
| Expansion 1 LV Day 21 | 1.0000 | 0.249 | | 0.0043 | 0.0011 | | 0.1071 | 110000000 | | 117777 |
| Expansion 2  No LV Day 21 | 1.0000 | 0.358 | | 0.0126 | 0.0045 | | 0.4511 | 183000000 | | 825476 |
| Expansion 2 LV Day 21 | 0.9990 | 0.321 | | 0.0203 | 0.0065 | | 0.651 | 183000000 | | 1191290 |
| Expansion 3 No LV Day 21 | 1.0000 | | 0.262 | 0.009 | 0.0024 | 0.2358 | | | 195000000 | 459810 |

LV stands for lentivirus added to the cells during expansion.

Supplementary Table 2: Results for flow cytometric analysis of spleen of liveborn SCID piglets

| Pig ID | Category of Humanization | Total human in all CD45+ leukocytes (%) | %CD3+ (T) | | %CD+ CD4+  (T helper) | %CD3+ CD8+  (T cytotoxic) | %CD3+ CD4+ CD8+ (Double Positive) | %CD19+ (B) | %CD163+ (Myeloid) |
| --- | --- | --- | --- | --- | --- | --- | --- | --- | --- |
| 18-1 | Humanized | 37789 (43.49) | | 80.90 | 0.25 | 21 | 0.10 | 5.40 | 0.25 |
| 19-2 | Humanized | 16937 (42.21) | | 63 | 3.20 | 19.50 | 1.20 | 7.90 | 0 |
| 27-1 | Humanized | 43240 (28.10) | | 59.70 | 35.90 | 23.90 | 2.23 | 15.90 | 24.10 |
| 27-3 | Humanized | 10078 (15.40) | | 66.20 | 43.70 | 29.40 | 2.04 | 6.72 | 26.90 |
| 30-1 | Humanized | 23678 (15.60) | | 27 | 56 | 14.70 | 2 | 21.40 | 71.40 |
| 30-3 | Very low | 1536 (1.05) | | 0 | 0 | 0 | 0 | 33.30 | 66.70 |
| 27-5 | Very low | 224 (0.23) | | 5.36 | 0 | 66.70 | 0 | 6.25 | 88.40 |
| 27-6 | Injected, no human cells | 0 (0) | | 0 | 0 | 0 | 0 | 0 | 0 |
| 27-7 | Very low | 8155 (2.32) | | 82 | 23.80 | 60.70 | 5.79 | 0.37 | 17.30 |
| 31-1 | Injected, no human cells | 0 (0) | | 0 | 0 | 0 | 0 | 0 | 0 |
| 31-4 | Very low | 216 (0.25) | | 0 | 0 | 0 | 0 | 50 | 50 |
| 19-1 | Injected, not humanized | 0 (0) | | 0 | 0 | 0 | 0 | 0 | 0 |

In the column for total human CD45+ cells, the total such cells detected is provided, followed by the percentage of total CD45+ cells in parentheses. In each succeeding column, the number shown is the % of cells positive for the human marker protein or protein combination shown. Predicted cell type is shown in parentheses in headers.

**Supplementary Table 3**: Results for flow cytometric analysis of bone marrow of liveborn SCID piglets

| **Pig ID** | **Category of Humanization** | **Total human in all CD45+ leukocytes (%)** | **%CD3+ (T)** | **%CD+ CD4+** | **%CD3+ CD8+** | **%CD3+ CD4+ CD8+ (Double Positive)** | **%CD19+ (B)** | **%CD163+ (Myeloid)** |
| --- | --- | --- | --- | --- | --- | --- | --- | --- |
|  |  |  |  | **(T helper)** | **(T cytotoxic)** |  |  |  |
| **18-1** | Humanized | 5355 (6.09) | 1.40 | 0.06 | 36 | 0.01 | 3.40 | 0 |
| **19-2** | Humanized | 5916 (6.58) | 11.10 | 48 | 39 | 12.90 | 62.30 | 0.44 |
| **27-1** | Humanized | 32957 (6.79) | 8.02 | 40.50 | 37.40 | 2.76 | 73.50 | 18.40 |
| **27-3** | Humanized | 12860 (2.96) | 15.50 | 45 | 34.60 | 2.06 | 57.70 | 26.80 |
| **30-1** | Humanized | 8695 (7.67) | 8.04 | 43 | 36.70 | 20.30 | 70.30 | 21.70 |
| **30-3** | Humanized | 1298 (0.27) | 0 | 0 | 0 | 0 | 22.20 | 0 |
| **27-5** | Very low | 73 (0.01) | 0 | 52.40 | 0 | 0 | 88.90 | 45.20 |
| **27-6** | Injected, no human cells | 0 (0) | 0 | 0 | 0 | 0 | 0 | 0 |
| **27-7** | Very low | 521 (0.74) | 16.70 | 52.40 | 32.30 | 8.35 | 77.10 | 21.10 |
| **31-1** | Injected, no human cells | 0 (0) | 0 | 0 | 0 | 0 | 0 | 0 |
| **31-4** | Very low | 88 (0.14) | 25 | 0 | 0 | 0 | 25 | 50 |
| **19-1** | Injected, not humanized | 0 (0) | 0 | 0 | 0 | 0 | 0 | 0 |

In the column for total human CD45+ cells, the total such cells detected is provided, followed by the percentage of total CD45+ cells in parentheses. In each succeeding column, the number shown is the % of cells positive for the human marker protein or protein combination shown. Predicted cell type is shown in parentheses in headers.
